# White Matter Connectivity as an Early Biomarker of Alzheimer's Disease: A Structural MRI and Network‐Based Study in Mild Cognitive Impairment with Neuropsychiatric Features

**DOI:** 10.1002/alz.091420

**Published:** 2025-01-09

**Authors:** Amir Mohammad pajavand, Michel J. Grothe, Michel Thiebaut de Schotten, Andrea Vergallo, Harald Hampel

**Affiliations:** ^1^ University of Tehran, College of Engineering, Tehran, Tehran Iran (Islamic Republic of); ^2^ Unidad de Trastornos del Movimiento, Servicio de Neurología Y Neurofisiología Clínica, Instituto de Biomedicina de Sevilla, Hospital Universitario Virgen del Rocío/CSIC/Universidad de Sevilla, Seville, Spain, Sevilla, Sevilla Spain; ^3^ Brain Connectivity and Behaviour Laboratory, Sorbonne University, Paris, France, Paris, Paris France; ^4^ Groupe d’Imagerie Neurofonctionnelle, Institut des Maladies Neurodégénératives‐UMR 5293, CNRS, CEA, University of Bordeaux, Bordeaux, France, Bordeaux, Bordeaux France; ^5^ Sorbonne University, GRC n° 21, Alzheimer Precision Medicine (APM), AP‐HP, Pitié‐Salpêtrière Hospital, Boulevard de l'hôpital, F‐75013, Paris France; ^6^ Sorbonne University, GRC n°21, Alzheimer Precision Medicine (APM), AP‐HP, Pitié‐Salpêtrière Hospital, Boulevard de L'hôpital, F‐75013, Paris France

## Abstract

**Background:**

Neuropsychiatric symptoms (NPS), including depression and circadian rhythm disruptions, are early non‐cognitive markers along the Alzheimer’s Disease (AD) continuum. These pathological states are thought to resemble AD pathogenesis, both of which are characterized by a marked decline in adult hippocampal neurogenesis.

**Method:**

96 elderly participants divided into three groups based on the global depression scale, neuropsychiatric inventory, clinical dementia rating, and mini‐mental status examination. Twelve MCI individuals with NPS (MCI+) and 49 without NPS (MCI‐) were classified, and 35 healthy individuals were matched for age and gender.

Voxel‐based morphometry and Tract‐Based Spatial Statistics were employed to conduct a comprehensive brain‐wide comparison between groups. We used a network‐based statistic method to probe deviations in brain structural WM connectivity.

**Result:**

The Hippocampus, Amygdala, and sensory cortex (SSC) exhibited GM loss in MCI+ versus healthy individuals. Furthermore, structural WM connectivity reductions in the thalamus, pallidum, putamen, caudate, hippocampus, SSC, and frontal cortex suggest the involvement of the limbic system before overt volumetric GM loss in the same group analyses.

**Conclusion:**

This study indicated structural WM connectivity loss within the Papez circuit before the GM integrity at the medial temporal lobe in individuals with MCI+ before their conversion to AD dementia. These findings shifted the focus to the Papez circuit during the prodromal phase of the disease, concurrent with pathological changes in the medial temporal lobe.

